# Road traffic injuries and deaths and the achievement of UN Sustainable Development Goals in Brazil: results from the Global Burden of Disease Study, 1990 to 2019

**DOI:** 10.1590/0037-8682-0261-2021

**Published:** 2022-01-28

**Authors:** Deborah Carvalho Malta, Otaliba Libânio de Morais, Laís Santos de Magalhães Cardoso, Guilherme Augusto Veloso, Fabiana Martins Dias de Andrade, Ana Maria Nogales Vasconcelos, Cheila Marina de Lima, Antonio Luiz Pinho Ribeiro, Mohsen Naghavi

**Affiliations:** 1 Universidade Federal de Minas Gerais, Departamento de Enfermagem Materno Infantil e Saúde Pública, Belo Horizonte, MG, Brasil.; 2 Universidade Federal de Goiás, Instituto de Patologia Tropical e Saúde Pública, Goiânia, GO, Brasil.; 3 Universidade Federal de Minas Gerais, Escola de Enfermagem, Programa de Pós-Graduação em Enfermagem, Belo Horizonte, MG, Brasil.; 4 Universidade Federal de Minas Gerais, Departamento de Estatística, Programa de Pós-Graduação em Estatística, Belo Horizonte, MG, Brasil.; 5 Universidade Federal de Minas Gerais, Faculdade de Medicina, Programa de Pós-Graduação em Saúde Pública, Belo Horizonte, MG, Brasil.; 6 Universidade de Brasília, Departamento de Estatística, Brasília, DF, Brasil.; 7 Ministério da Saúde, Secretaria de Vigilância em Saúde, Brasília, DF, Brasil.; 8 Universidade Federal de Minas Gerais, Faculdade de Medicina, Hospital das Clínicas, Belo Horizonte, MG, Brasil.; 9 University of Washington, Institute for Health Metrics and Evaluation, Seattle, Washington, USA.

**Keywords:** Traffic accidents, Global Burden of Disease, Mortality, Disability adjusted life years, Wounds and injuries

## Abstract

**INTRODUCTION:**

Brazil ranks 5th in the number of deaths due to road injuries. This study aimed to analyze mortality and disabilities resulting from road injuries in Brazil, and to assess the Sustainable Development Goals (SDG) target of reducing deaths due to road injuries by 50% by 2030.

**METHODS:**

This descriptive and exploratory study used the estimates from the Global Burden of Disease 2019: indicators of mortality, premature deaths, and disabilities according to sex, age group, and type of transport for 1990, 2015, and 2019. Time trends in mortality rates from 1990 to 2019 were assessed, and a projection for 2030 was calculated, applying a linear regression model.

**RESULTS:**

Deaths due to road injuries were 44,236 in 1990, and 44,529 in 2019, representing a 43% reduction in mortality rates. The highest rates were in the North, Northeast, and Midwest regions of Brazil, in males and young adults. A 77% reduction was observed in mortality rates for pedestrians and an increase of 53% for motorcyclists and of 54% for cyclists during the period. In terms of motorcycle road injuries, the mortality rate for men increased from 7.3/100,000 (1990) to 11.7/100,000 inhabitants (2019). The rates of premature deaths and disabilities were also higher for men when compared to women. Amputations, fractures, spinal cord injuries, and head trauma were the main types of road injuries. The projections for 2030 show that Brazil might not reach the SDG target.

**CONCLUSIONS:**

Despite the decline in mortality rates, the 2030 Agenda’s target might not be achieved.

## INTRODUCTION

Road traffic injuries are responsible for approximately 1.35 million deaths around the world and for approximately 50 million non-fatal injuries, and in most cases, victims are left disabled^1^ ^,^ ^2^. The risk of death due to road traffic injuries is unevenly distributed throughout the world; in low income countries, the average death rate is 27.5/100,000 inhabitants, which is three-fold higher than the rate in high-income countries, where the average rate is 8.3/100,000 inhabitants^1^.

Road traffic injuries are the main cause of death for children and young people aged 5 to 29 years, most of which happen to men^1^. Globally, pedestrians and cyclists correspond to approximately one fourth of the fatal victims of road traffic injuries^2^. In Brazil, road traffic injuries cause approximately 40,000 deaths each year, and the country ranks 5th in number of deaths^3^. If we include the seriously injured, the number of victims is over 150,000/year^4^. Although the mortality rates have been stable in state capitals, there is an upward trend in smaller towns, with great variability in rates among the states^3^. 

In February 2020, the 3rd Global Ministerial Conference on Road Safety brought together governments and community representatives from more than 140 countries, and restated one main objective of the Sustainable Development Goals (SDGs), which is to reduce deaths due to road injuries by 50% by 2030^2^. Therefore, it is essential that countries monitor the evolution of morbimortality caused by road traffic injuries. Taking this into consideration, the objectives of the current study are to analyze mortality and disabilities resulting from road traffic injuries in Brazil, and to estimate the trends of mortality rates due to road injuries from 1990 to 2019, and their projection for 2030.

## METHODS

This is an epidemiological study with a descriptive and exploratory approach, in which GBD 2019 estimates for Brazil and its states, produced by the Institute of Health Metrics Evaluation (IHME), from the University of Washington, were used. With each edition, new data and methods are incorporated, and the estimations are updated for the analyzed period. Sources of data and methods are described elsewhere^5^ ^,^ ^6^. The main source of information used by the GBD to estimate mortality in Brazil was data from the Mortality Information System (SIM, in Portuguese), from the Brazilian Ministry of Health (MS).

These estimates consider the correction of the under-reporting of deaths and the redistribution of ill-defined and imprecise causes (*garbage codes*), which stem from statistical modelling detailed by age, sex, year, and cause of death^6^ ^,^ ^7^. The GBD estimates are presented with the 95% uncertainty intervals^8^ (95% UI) due to the uncertainties of all sources and modelling steps, as well as the variability in sample size, among other reasons^9^.

The GBD 2019 is based on the International Statistical Classification of Diseases (ICD) 9 and 10, and organizes the basic causes of death in a four-level hierarchy. Level 1 has 3 large groups of diseases: communicable, maternal, neonatal, and nutritional; non-communicable; and injuries. Level 2 divides these groups into 21 causes. Level 3 establishes distinctive causes for 168 diseases. Level 4 separates these into 369 causes. For the current study, levels 3 and 4 were used, which allow us to disaggregate transport injuries and their typology. The estimates of injury incidence and deaths due to injury are based on the codes E000-E999 of the ICD-9, and codes V01 to Y98 of chapter XX of the ICD-10^6^. More information on the GBD list of injury codes and categories used to estimate the indicators is published elsewhere^6^. GBD also uses algorithms to redistribute deaths from unspecified codes (garbage codes) to specific categories of causes in order to correct for known bias^6^.

Data for Brazil and its states from 1990, 2015, and 2019 were compared. The data were explored according to sex, age group, and types of road transport (pedestrians, cyclists, automobile occupants, motorcyclists, other types of land transport). In 2019, the number of deaths, age-standardized mortality rates, disability adjusted life years (DALY), years of life lost (YLL) due to premature death, and years lost due to disability (YLD) rates were analyzed by state^5^ ^,^ ^7^ ^,^ ^9^.

Our study presented the prevalence of disability caused by road injuries estimated by the GBD, whose calculation method is described in detail elsewhere^5^. Disabilities are calculated by the IHME using the DisMod-MR 2.1, an epidemiological descriptive meta-regression tool. Road injury incidence data, obtained from emergency rooms records and hospital admissions, were used to estimate the incidence by place, year, age group, and sex for each type of road transport. The estimates of incidence were converted into prevalence, using the average duration of each type of injury, according to the Dutch Legion Surveillance System^10^ and based on the literature^5^. Therefore, the IHME estimates the probability of YLD according to the prevalence of conditions, such as spinal cord injuries, head trauma, and other injuries, multiplying the prevalence of the injuries by the weights for previously mapped disability^11^ ^,^ ^12^. 

Finally, when considering the SDG target 3.6, which refers to the reduction of mortality rates due to road injuries by 50% by 2030^2^, projections of the age-standardized mortality rates were carried out for this time frame. The linear regression model was applied to perform the projections considering different segments of the historical series under study: from 2015 to 2019, 2000 to 2019, and 1990 to 2019. 

This project was approved by the Research Ethics Committee of Universidade Federal de Minas Gerais (UFMG), logged under protocol number CAAE: 62803316.7.0000.5149.

## RESULTS

There were 134,642 deaths due to injuries (self-harm and violence, transport injuries, and unintentional injuries combined) in 1990 (95% UI: 131,249-138,569), 172,320 in 2015 (95% UI: 168,132-176,598), and 168,284 in 2019 (95% UI: 162,167-174,330) (Supplementary Material 1A). Deaths caused by road traffic injuries were 44,236 in 1990, 46,729 in 2015, and 44,529 in 2019. The mortality rates for this type of cause were 32.4 (32.2-34.6) in 1990, 21.2 (20.7- 21.8) in 2015, and 19.1 (18.2-19.9) in 2019. Therefore, a 43% reduction in the rates was found from 1990 to 2019 (Supplementary Material 1B). The mortality rate for pedestrians in 2019 was 5.1/100,000 inhabitants. For automobile drivers, 6.4/100,000; for motorcyclists, 6.5/100,000; for cyclists, 0.8/100,000; and for other road vehicles, 0.3/100,000. The rates according to the type of road transport had a considerable variation from 1990 to 2019: a reduction of 77% was found in mortality rates for pedestrians; while there was an increase for motorcyclists and cyclists, with a percentage variation of 53% and 54%, respectively (Supplementary Material 1B). 

Table 1 shows the number of deaths, mortality rates, DALYs, YLLs, and YLDs due to road injuries in 2019. The states from the North and Northeast regions had the highest mortality rates due to road traffic injuries in 2019, especially Tocantins (30.9; 95% UI: 25.7-36.3), Rondônia (28.8; 95% UI: 24.6-33.4), Roraima (27.8; 95% UI: 24.7-30.8), and Piauí (27.7; 95% UI: 24.6-31.3). In addition to these, three more states of the Northeast (Ceará, Maranhão, and Sergipe), two from the Midwest (Mato Grosso and Goiás), and one from the Southeast are part of the 10 states with the highest mortality rates due to road injuries. The lowest rates per 100,000 inhabitants were verified in: Amazonas (13.5; 95% UI: 11.9-15.2), São Paulo (13.9; 95% UI: 12.5-15.5), Distrito Federal (15.6; 95% UI: 14.1-17.5), Rio de Janeiro (15.7; 95% UI: 14.1-17.4), and Rio Grande do Sul (15.7; 95% UI: 14.1-17.7). The same states from the Northeast, North, and Midwest regions had the highest rates of DALYs, YLDs (disability), and YLLs (premature death) per 100,000 inhabitants in 2019 (Table 1). 

**TABLE 1:** Number of deaths, mortality rates, DALY, YLD, and YLL rates due to road traffic injuries, in Brazil and its states, and respective 95% uncertainty intervals, 2019, GBD 2019.

State	Number of Deaths			Mortality Rate*			DALY Rate*			YLD Rate*			YLL Rate*		
	n	95% UI		Value	95% UI		Value	95% UI		Value	95% UI		Value	95% UI	
		Lower	Upper		Lower	Upper		Lower	Upper		Lower	Upper		Lower	Upper
Brazil	44529	42510	46388	19.1	18.3	20.0	1061	1002	1114	124	90	164	937	890	979
Acre	143	126	160	16.3	14.4	18.2	895	799	994	120	88	158	775	687	873
Alagoas	855	747	977	23.3	20.4	26.6	1244	1101	1409	119	86	158	1126	990	1281
Amapá	134	120	149	16.7	15.0	18.4	951	856	1049	164	119	217	787	707	867
Amazonas	536	473	608	13.5	11.9	15.2	712	638	797	81	59	108	631	560	712
Bahia	3133	2591	3716	18.7	15.5	22.1	959	819	1112	100	73	132	859	717	1014
Ceará	2718	2231	3266	26.1	21.4	31.2	1410	1190	1665	146	106	195	1264	1048	1514
Distrito Federal	482	431	544	15.6	14.1	17.5	816	737	907	102	74	137	714	643	800
Espírito Santo	1075	939	1215	24.9	21.8	28.2	1369	1214	1528	150	108	200	1220	1070	1382
Goiás	1969	1650	2322	26.5	22.3	31.1	1424	1216	1641	159	115	211	1265	1067	1481
Maranhão	1932	1592	2304	24.8	20.4	29.7	1303	1102	1519	169	121	223	1134	940	1356
Mato Grosso	1036	924	1154	27.2	24.3	30.2	1526	1369	1682	180	131	238	1346	1203	1496
Mato Grosso do Sul	651	575	732	21.4	18.9	24.0	1212	1075	1361	182	133	240	1030	899	1161
Minas Gerais	4461	3974	4962	18.7	16.7	20.8	1031	926	1139	106	77	142	925	822	1030
Pará	1761	1564	1987	19.4	17.2	21.8	1037	930	1158	117	86	155	920	816	1040
Paraíba	1042	898	1208	22.9	19.8	26.5	1276	1118	1454	128	93	169	1148	996	1323
Paraná	2924	2584	3292	23.5	20.9	26.4	1293	1157	1445	122	89	162	1171	1040	1322
Pernambuco	2318	2028	2633	21.9	19.3	24.9	1178	1045	1328	110	79	146	1068	937	1211
Piauí	1048	929	1183	27.7	24.6	31.3	1555	1382	1740	171	124	227	1383	1219	1564
Rio de Janeiro	3086	2766	3429	15.7	14.1	17.4	897	806	987	127	92	169	770	686	853
Rio Grande do Norte	712	585	847	18.0	14.8	21.4	977	823	1137	97	71	129	880	729	1037
Rio Grande do Sul	2007	1796	2263	15.7	14.1	17.7	875	790	972	101	74	134	774	695	868
Rondônia	537	457	624	28.8	24.6	33.4	1557	1357	1761	188	137	248	1369	1174	1575
Roraima	157	139	174	27.8	24.7	30.8	1527	1370	1693	219	159	289	1308	1159	1452
Santa Catarina	1764	1583	1976	22.6	20.4	25.3	1272	1152	1406	122	88	162	1150	1030	1282
São Paulo	6939	6199	7747	13.9	12.5	15.5	823	745	914	125	90	166	698	624	779
Sergipe	598	500	703	23.7	19.8	27.8	1295	1110	1488	130	95	172	1165	975	1363
Tocantins	512	427	602	30.9	25.7	36.3	1527	1276	1776	117	85	156	1410	1153	1660

**DALY:** disability adjusted life years; **YLD:** years lost due to disability; **YLL:** years of life lost; **95% UI:** 95% uncertainty intervals; *Age-standardized rate, both sexes, per 100,000 inhabitants.

**FIGURE 1:**
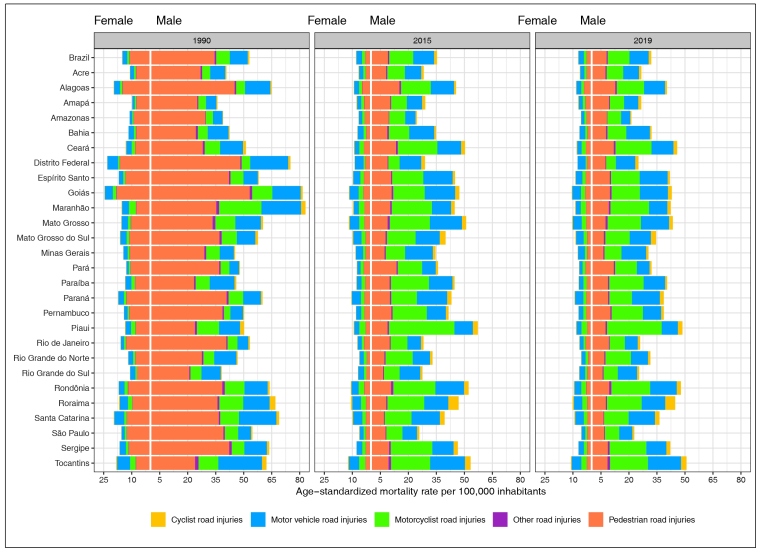
Age-standardized mortality rates due to road injuries by type of road transport, per 100,000 inhabitants, according to year (1990, 2014, and 2019), Brazil and states, and sex, GBD 2019.

Figure 1 and Supplementary Materials 2 and 3 show the standardized mortality rates for 1990, 2015, and 2019, by states and by sex, according to the type of road transport. For Brazil, a reduction in the mortality rates was observed for pedestrians during the period. For women, those rates went from 11.0 in 1990 to 3.1 and 2.6 in 2015 and 2019, respectively. For men, in the same years, the rates went from 34.4 to 9.1 and to 8.0 per 100,000 inhabitants. For automobile occupants, the rates for women increased from 2.5 (1990) to 2.8 (2019) and for men, from 9.6 (1990) to 10.2 (2019). An increase in mortality rates was also found for motorcyclists. For women, these rates rose from 1.2/100,000 inhabitants in 1990 to 1.5 in 2019; and for men, from 7.3 in 1990 to 11.7 in 2019. Death rates for cyclists also increased. For the occupants of other types of vehicles, the rates remained stable (Figure 1 and Supplementary Materials 2 and 3).

**FIGURE 2:**
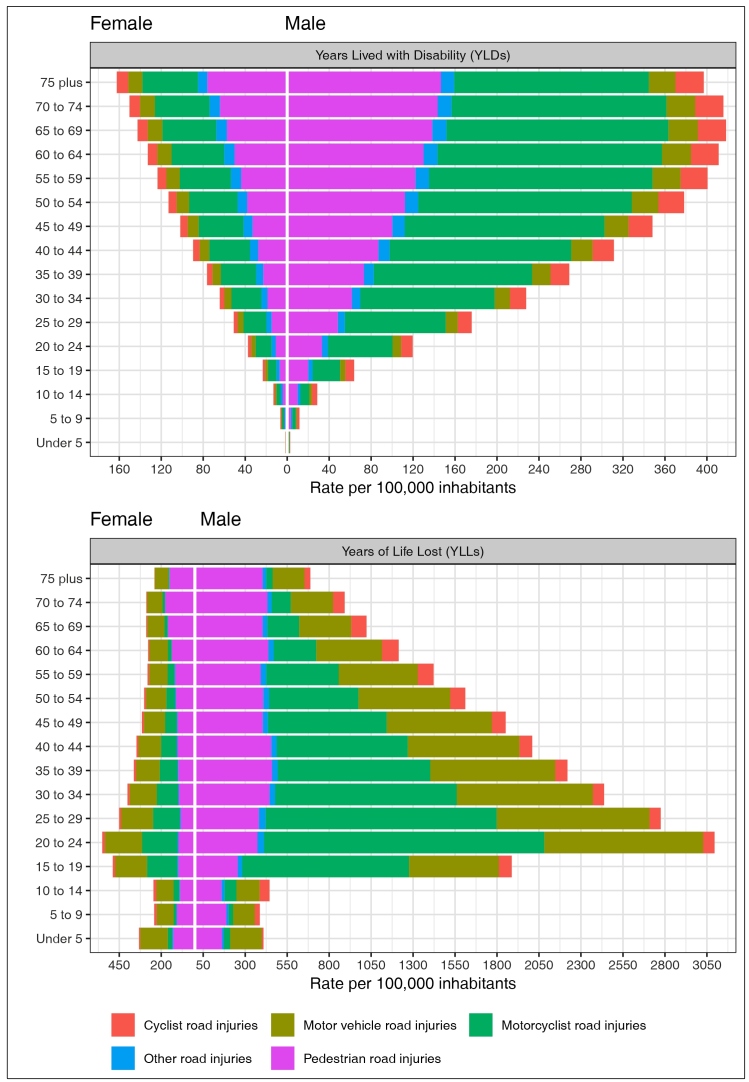
YLD and YLL rates due to road injuries by type of road transport, per 100,000 inhabitants, according to age group and sex, Brazil 2019, GBD 2019. Legend: **YLL:** years of life lost; **YLD:** years lost due to disability.

Figure 2 shows the YLL rates, referring to premature deaths, and YLD rates, referring to disability caused by road traffic injuries in 2019. Overall, the YLL and YLD rates were higher for men when compared to women. In terms of YLD for both sexes, the rates were higher among motorcyclists and pedestrians, especially for the age group of 60 to 74 years of age. Concerning the YLL indicator, premature mortality among men was higher for motorcyclists, for occupants of motor vehicles, and for pedestrians in the younger age groups (20 to 29 years of age). Among women, the YLL rates due to road injuries for motorcyclists and for occupants of motor vehicles stand out within the age group of 15 to 24 years and for pedestrians of 60 years and older (Figure 2).

**FIGURE 3:**
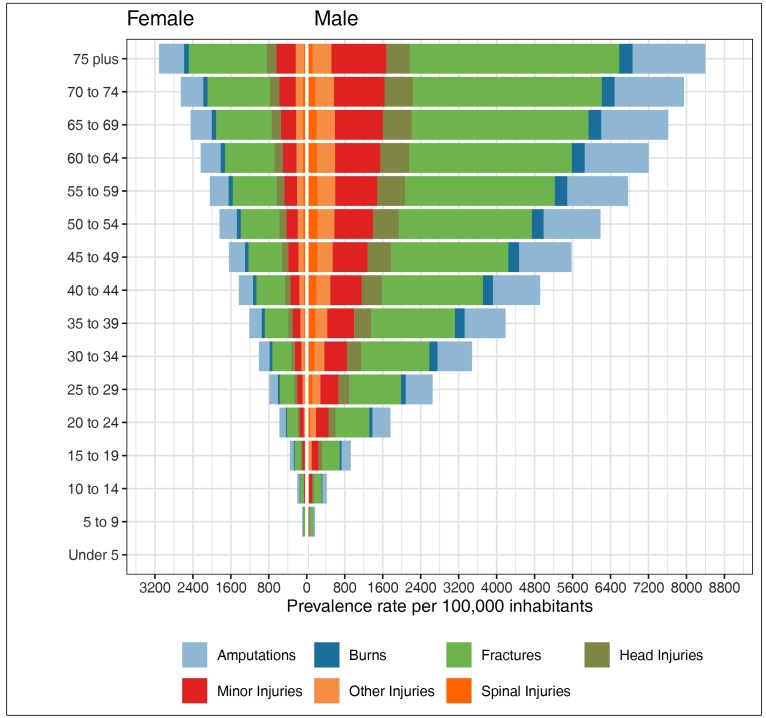
Prevalence rates of the types of injuries caused by road traffic accidents, per 100,000 inhabitants, according to age group and sex, Brazil, 2019, GBD 2019

Figure 3 shows the prevalence of different types of road traffic injuries, according to age group and sex, for 2019. Fractures and amputations were the most frequent for both sexes. For men, the number of fractures was high in all of the age groups, and was highest in the elderly age group. Spinal cord and head injuries, which are more serious and can cause definitive disabilities, were more frequent in men and happened in every age group (Figure 3).

**FIGURE 4:**
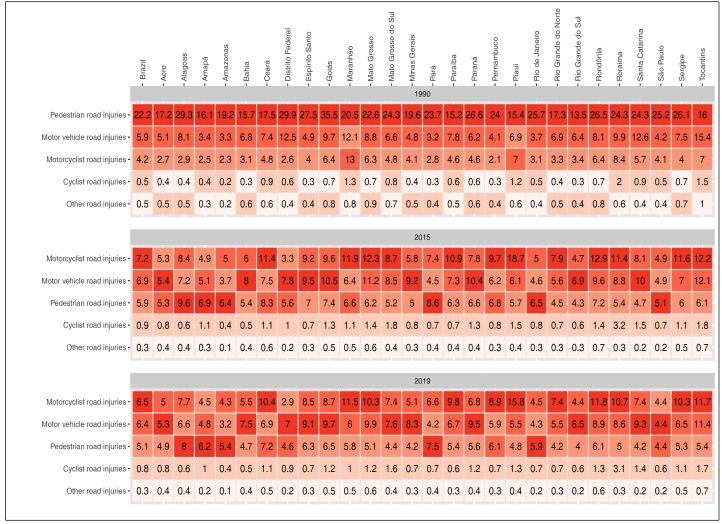
Age-standardized mortality rates due to road traffic injuries by type of road transport (motorcyclists, occupants of automobiles, pedestrians, cyclists, occupants of other road transport vehicles), per 100,000 inhabitants, both sexes, according to year (1990, 2014, and 2019), Brazil and its states, GBD 2019.

The analysis of the risk of death in the states, in 1990, revealed a higher risk for pedestrians, especially in the states Goiás (35.5 per 100,000 inhabitants), Distrito Federal (29.9), Alagoas (29.9), Paraná (26.6), Rondônia (26.5), Rio de Janeiro (25.7), São Paulo (25.2), and Sergipe (26.1). The second highest risk was for the occupants of automobiles and motorcyclists. In 2015, the mortality rates for motorcyclists were the highest risk in 13 of the 27 states. In 2019, mortality of motorcyclists ranked the highest in 12 states, especially Piauí (15.8 per 100,000 inhabitants). Mortality for occupants of automobiles ranked first in 11 states, with the highest rates in Goiás (9.7), Paraná (9.5), and Espírito Santo (9.1). Pedestrian death rates were the highest in Alagoas (8), Pará (7.5), Amapá (6.2), Rio de Janeiro (5.9), and Amazonas (5.4) (Figure 4). 

**FIGURE 5:**
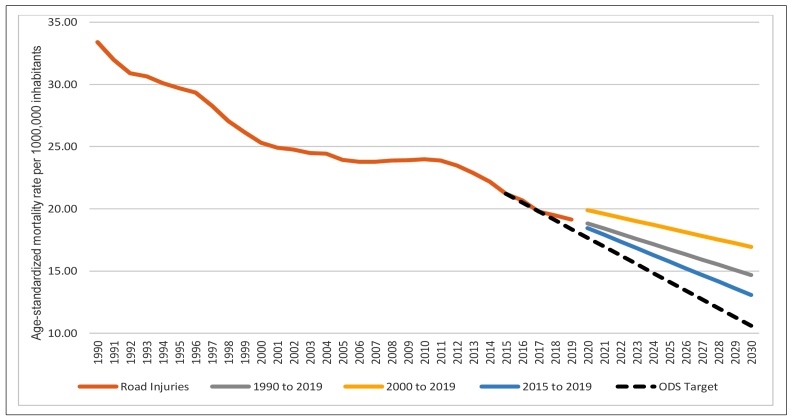
Trends of the age-standardized mortality rates due to road traffic injuries, both sexes, from 1990 to 2019, and projections of the rates for 2030, according to trends of time periods (1990 to 2019, 2000 to 2019, 2015 to 2019), Brazil, GBD 2019. Legend: **SDG:** Sustainable Development Goal.

In terms of achieving the SDG target of reducing the age-standardized mortality rates due to road traffic injuries by 50%, the projections for 2030 in all the evaluated sets of years show that Brazil will not reach that goal. The best result was observed when only the rates of the recent years (2015 to 2019) were considered in the prediction model (Figure 5).

## DISCUSSION

The present study shows a reduction in death rates due to road traffic injuries from 1990 to 2019, mostly in the period from 1990 to 2015; however, this reduction was uneven among regions. The rates were higher in the states of the North, Northeast, and Midwest regions. One can notice a change in the magnitude of the risk of death for different groups, with a significant reduction in pedestrian deaths and an increase in motorcyclist and cyclist deaths. Premature deaths were more frequent among young males, and the sum of disabilities over the years showed a greater prevalence among the elderly. Projections reveal that the SDG target number 3.6 might not be reached. 

Higher death rates were observed among young males, which is consistent with national and international literature^1^ ^,^ ^13^ ^,^ ^14^. Studies show a consistent prevalence of males over females in terms of road traffic injuries. Sex differences in mortality rates are most likely related to the fact that men have greater exposure and are more likely to engage in risky behavior^14^. In the context of a chauvinistic culture, which encourages male power and dominance, a car is a symbol of power and freedom. Driving at high speed is an opportunity to challenge and overcome the limits established for safe behavior^15^, which commonly results in more accidents and higher rates of injuries caused by road traffic accidents^15^. 

Global studies show that more than 50% of deaths occur among young adults, 15 to 44 years of age^14^. It is important to emphasize that road injuries are the second most important cause of death among children and adolescents throughout the world^2^ ^,^ ^14^. In the present analysis, the relevance of premature deaths (YLL), as well as that of disabilities (YLD), stands out, especially among young people, but also among the elderly.

The present study reveals the relevance of amputations, fractures, head trauma, and spinal cord injuries as the cause of disabilities due to road accidents. Studies conducted in Brazil estimate that hospitalization with a diagnosis suggesting lasting physical sequelae represent ¼ of all hospitalizations due to road accidents, and these occur mostly among young males and motorcyclists^16^.

The elderly, especially pedestrians, have very high rates of injuries and deaths on the streets. This occurs because of the increased fragility due to advanced age, more prevalence of osteoporosis, walking difficulties, and a reduction in visual and auditory acuity. This is compounded by the poor conditions of the sidewalks, which are uneven; the lack of signs; and traffic lights with insufficient time for pedestrian crossing^2^ ^,^ ^6^. As drivers, the elderly are also frequent victims. The need for mobility exposes them to a greater risk. Physical limitations related to advanced age can affect the ability to drive, to react quickly, to brake, among other reflexes^2^ ^,^ ^14^. The present study shows that the DALY and disability rates were high among the elderly, which underlines the high burden of the consequences of the disabilities caused by road traffic injuries throughout life.

Regarding trends, this study showed a reduction in mortality rates, although still insufficient to reach target number 3.6 of the SDG. This can be explained by a series of determining factors, the most important of which is traffic legislation, especially the Brazilian Traffic Code (CTB, in Portuguese), which is the main traffic regulation in the country, in place since 1998, and which has undergone several changes over the years. The CTB introduced new legislation and established municipal authority in terms of traffic regulations; it has also created many protective measures, especially the regulation of speed limits for different types of traffic routes, the prohibition of driving under the influence of alcohol, the mandatory use of helmets for motorcyclists, the mandatory use of seatbelts, and child safety equipment for cars^17^.

Although there has been a reduction in mortality rates in practically every state, with the exception of Piauí, the variation is very high. More accentuated decreases were found in the South and Southeast regions, which might be explained by a more efficient implementation of the CTB, as exemplified by a better enforcement of speed limits and the prohibition of driving under the influence of alcohol^13^. Also important were improvements in street infrastructure, a better fiscalization of mandatory safety items (seatbelts, child safety features), the use of ABS brakes, and more traffic education campaigns^3^ ^,^ ^18^.

Other studies identify differences in the risk of road traffic injuries and deaths according to regions and to population size. A decline in deaths was observed in the South and Southeast regions and in cities with large populations, while a rise was found in the Northeast and Midwest regions, and in small, countryside towns as well^3^. Those differences can be explained by the decentralization of traffic regulation enforcement, which was transferred from state level to municipal level. That, however, has occurred in an unequal manner in recent decades. Each state capital has undergone traffic enforcement decentralization^3^ ^,^ ^19^, but only 28.6% of the smaller towns (not capitals) have undergone similar changes^3^. The most affected states are in the North and Northeast regions, which have a large number of poor towns with less ability to enforce regulations and inspect traffic^3^. 

The differences in the trends according to the type of road transport should also be highlighted. The decline in deaths among pedestrians and occupants of automobiles is consistent with the international trend^6^, and can be related to inspection, seat belts, child safety equipment, improvements in traffic signs, and improvements in safety features for vehicles, as well as to actions of traffic safety education^3^ ^,^ ^20^.

The rise in mortality rates of motorcyclists during this period, especially after 2000, has also been observed by other studies conducted in Brazil^13^ ^,^ ^21^ ^-^ ^23^. This may well be connected to a growth in the number of motorcycles, especially in states of the Northeast region, which witnessed an increase of 1,400% in motorcycle sales between 1991 and 2008^24^. That increase is related to the growth in economic activity between 2004 and 2014, and the growth in the number of motorcycles, which have been used for work in both the urban and rural environments^3^ ^,^ ^22^, even substituting more traditional forms of transport, such as bicycles and animals^13^.

Mortality rates among cyclists have increased due to a higher exposure and increased use of bicycles, in response to the encouragement of healthy and sustainable habits. Bicycles are an economical, agile, and affordable type of transport. This is also a more active type of transport, which brings health benefits to the people and is an option for leisure activity^14^. However, it is necessary to provide safety to cyclists by implementing such measures as bicycle lanes, investments in traffic education, the awareness of drivers to a more respectful attitude towards cyclists, adequate signaling, the use of helmets, among others^14^.

Other public policies implemented in the country add to the CTB and contribute to reducing mortality by road injuries. Among these are the “Dry Law”^25^ and the “New Dry Law”^26^, from 2008 and 2012, respectively, which enabled better controls against driving under the influence of alcohol. Also important was the implementation of the Mobile Emergency Care Service (SAMU, in Portuguese) in 2004^27^, which had an impact in terms of improving the response to emergencies. Moreover, in 2010, the Life in Traffic project was created in a partnership joining the Health Ministry, the Pan American Health Organization (PAHO), and the World Health Organization (WHO)^28^. This project was an advancement in terms of improving the traffic safety policy governance, as well as the integration and qualification of information on health and traffic safety. It also allowed for interventions in a more articulated, interconnected, and integrated manner^29^.

The projections for 2030 indicate that the country will not meet the SDG target number 3.6. Despite the major improvements in regulations and the preservation of life that have been achieved since 1998, there is still much to be done by the government and society in order to enable Brazil to reach the levels of safety in traffic seen in European countries^1^. Going against the trend of what has been proposed internationally, recent setbacks in traffic laws have been observed in Brazil. The changes in the CTB in 2020, allowing for more tolerance towards traffic violations and making it more difficult to suspend driving licenses, certainly compromise traffic safety^30^. Other actions by the government, such as discrediting the enforcement of speed limits and the proposal to make child seats for children under 7 years of age optional, will contribute to an increase in the risk of traffic injuries and deaths. 

The GBD results are important in terms of standardizing methods, enabling comparisons between states and allowing for the analysis of evolution over time, and in so doing, it provides a more accurate understanding of the health problems in the country and improves health planning actions. However, among its limitations, we must highlight that the disability estimates of the GBD are based on international literature; therefore, there may well be differences between countries which have not been noted. Moreover, the GBD estimates of mortality come from the SIM, and it is well-known that there is an under-reporting of cases and a high proportion of undetermined external causes (violence and accidents) that can result in redistribution errors. 

## CONCLUSION

The results of this study show a decline in the mortality rates due to road traffic injuries from 1990 to 2019. There was a decline in pedestrian deaths and a rise in deaths of motorcyclists and cyclists. Projections indicate that the target of reducing death rates due to road traffic injuries by 50% until 2030 will not be reached. There are regional inequalities, which may be related to the uneven implementation of the CTB in all municipalities, differences in enforcement effectiveness, as well as incongruences in the effectiveness of educational measures. The positioning of the government and the loosening of the regulations might make it even more difficult to meet the SDG target.
