# Supplementary material for: Road traffic injuries and deaths and the achievement of UN Sustainable Development Goals in Brazil: results from the Global Burden of Disease Study, 1990 to 2019
Source: Rev Soc Bras Med Trop. 2022 Jan 28;55(Suppl 1):e0261-2021. doi: 10.1590/0037-8682-0261-2021 (PMC9038143; doi:10.1590/0037-8682-0261-2021)
Supplement: Supplementary file 1 [file 1678-9849-rsbmt-55-s01-e0261-2021-supp1.pdf]

SUPPLEMENTARY MATERIAL

Injuries (self-harm and violence, transport injuries, and unintentional injuries combined) were responsible for 134,642 (95% UI: 131,249-138,569) deaths in 1990, 172,320 (95% UI: 168,132-176,598) in 2015 and 168,284 (95% UI: 162,167-174,330) in 2019 (supplementary material 1A). The mortality rates due to injuries decreased from 100.9 (95% UI: 98.3-103.6) to 79.5 (95% UI: 77.4-81.6) and 73.7 (95% UI: 70.8-76.4) per 100,000 inhabitants, in 1990, 2015, and 2019, respectively; a reduction of 27% between 1990 and 2019 (supplementary material 1B). Deaths due to road traffic injuries account for approximately 30% of all deaths due to injuries (supplementary material 1A).

In terms of type of road transport, a 77% reduction was observed in the percentage of pedestrian mortality rates during the period (1990 to 2019). The rates were 22.2, 5.9, and 5.1 deaths per 100,000 inhabitants in 1990, 2015, and 2019, respectively. For motorcyclists and cyclists, an increase in mortality rates was observed, with percentage variations of 53% and 54% in 1990 and 2019, respectively (supplementary material 1B).

Supplementary Material 1A – Number of deaths by all causes, injuries, transport injuries, road traffic injuries and types of road transport, both sexes, according to year, and percentage variations between 1990 and 2015, 2015 and 2019, 1990 and 2019, Brazil, GBD 2019 .

Cause	Number of Deaths					
	1990	2015	2019	Percent Change		
	n (95% UI)	n (95% UI)	n (95% UI)	1990-2015	2015-2019	1990-2019
i) All causes	984030 (961703 - 1006977)	1319520 (1300260 - 1340710)	1411016 (1375412 - 1448298)	34%	7%	43%
ii) Injuries	134642 (131249 - 138569)	172320 (168132 - 176598)	168284 (162167 - 174330)	28%	-2%	25%
ii.1) Transport injuries	46327 (44725 - 47927)	48417 (47212 - 49639)	46174 (44092 - 48070)	5%	-5%	0%
ii.1.1) Road injuries	44236 (42663 - 45797)	46729 (45562 - 47901)	44529 (42510 - 46388)	6%	-5%	1%
ii.1.1.1) Pedestrian road injuries	28561 (27514 - 29740)	12594 (12211 - 12964)	11884 (11201 - 12528)	-56%	-6%	-58%
ii.1.1.2) Motor vehicle road injuries	8139 (7744 - 8481)	15309 (14905 - 15722)	14886 (14221 - 15549)	88%	-3%	83%
ii.1.1.3) Motorcyclist road injuries	6156 (5842 - 6446)	16202 (15779 - 16666)	15139 (14257 - 15839)	163%	-7%	146%
ii.1.1.4) Cyclist road injuries	712 (633 - 785)	1890 (1821 - 1958)	1872 (1760 - 1990)	166%	-1%	163%
ii.1.1.5) Other road injuries	668 (597 - 723)	734 (702 - 768)	749 (703 - 797)	10%	2%	12%
ii.1.2) Other transport injuries	2091 (1995 - 2205)	1688 (1625 - 1745)	1645 (1551 - 1730)	-19%	-3%	-21%
