# Supplementary material for: Road traffic injuries and deaths and the achievement of UN Sustainable Development Goals in Brazil: results from the Global Burden of Disease Study, 1990 to 2019
Source: Rev Soc Bras Med Trop. 2022 Jan 28;55(Suppl 1):e0261-2021. doi: 10.1590/0037-8682-0261-2021 (PMC9038143; doi:10.1590/0037-8682-0261-2021)
Supplement: Supplementary file 3 [file 1678-9849-rsbmt-55-s01-e0261-2021-supp3.pdf]

Supplementary Material 2 – Age-standardized mortality rates per 100,000 inhabitants, by type of road transport according to state, year and sex, GBD 2019.

State	Cyclist road injuries						Motor vehicle road injuries						Motorcyclist road injuries						Other road injuries						Pedestrian road injuries					
	1990		2015		2019		1990		2015		2019		1990		2015		2019		1990		2015		2019		1990		2015		2019	
	F	M	F	M	F	M	F	M	F	M	F	M	F	M	F	M	F	M	F	M	F	M	F	M	F	M	F	M	F	M
Brazil	0.2	0.9	0.2	1.6	0.2	1.4	2.5	9.6	3.0	11.2	2.8	10.2	1.2	7.3	1.6	12.9	1.5	11.7	0.2	0.9	0.1	0.6	0.1	0.6	11.0	34.4	3.1	9.1	2.6	8.0
Acre	0.2	0.6	0.3	1.3	0.3	1.3	2.1	8.0	2.3	8.7	2.2	8.4	1.0	4.4	1.2	9.4	1.2	8.9	0.2	0.8	0.1	0.7	0.1	0.6	7.6	26.9	2.8	8.1	2.5	7.4
Alagoas	0.2	0.7	0.2	1.1	0.2	1.1	3.2	13.5	2.6	12.4	2.5	11.2	1.3	4.8	1.6	16.0	1.5	14.7	0.2	0.8	0.1	0.8	0.1	0.8	14.8	45.1	4.7	15.2	4.0	12.7
Amapá	0.2	0.6	0.4	1.7	0.4	1.6	1.2	5.5	2.2	8.1	2.1	7.6	0.8	4.1	1.5	8.4	1.4	7.7	0.1	0.5	0.1	0.4	0.1	0.4	7.5	25.1	3.6	10.3	3.2	9.3
Amazonas	0.1	0.3	0.2	0.7	0.2	0.7	1.4	5.1	1.8	5.5	1.7	4.9	0.7	3.9	1.3	8.6	1.1	7.4	0.1	0.3	0.0	0.2	0.0	0.2	8.9	29.4	3.3	9.5	2.7	8.2
Bahia	0.1	0.5	0.2	1.0	0.2	0.9	2.8	11.1	3.1	13.4	3.0	12.6	1.1	5.3	1.4	10.9	1.3	10.1	0.2	1.0	0.1	0.8	0.1	0.8	7.6	24.4	2.5	8.7	2.1	7.7
Ceará	0.3	1.6	0.3	2.0	0.3	2.0	3.0	12.3	2.6	12.8	2.4	11.8	1.7	8.2	2.3	21.1	2.1	19.3	0.2	1.1	0.1	1.1	0.1	1.0	8.1	28.0	3.8	13.3	3.1	11.8
Distrito Federal	0.1	1.3	0.1	2.0	0.1	1.8	5.7	20.3	4.7	11.5	4.3	10.3	0.8	4.6	0.8	6.1	0.7	5.2	0.1	0.7	0.1	0.4	0.0	0.4	16.3	48.2	3.1	8.9	2.5	7.4
Espírito Santo	0.1	0.5	0.2	1.3	0.2	1.2	2.3	7.7	3.7	15.7	3.5	15.0	1.1	7.0	1.9	16.7	1.8	15.4	0.1	0.8	0.1	0.6	0.1	0.6	13.3	42.1	3.7	10.8	3.1	9.8
Goiás	0.2	1.1	0.3	2.2	0.3	2.1	4.3	15.1	4.9	16.3	4.6	15.0	1.9	10.9	2.5	16.9	2.3	15.2	0.3	1.2	0.1	0.9	0.1	0.9	17.9	53.2	4.0	11.0	3.4	9.8
Maranhão	0.3	2.4	0.4	1.9	0.3	1.8	3.6	21.2	2.8	10.3	2.6	9.8	3.7	22.7	2.9	21.4	2.9	20.6	0.2	1.4	0.1	1.0	0.1	0.9	7.5	35.3	3.5	10.2	2.9	9.2
Mato Grosso	0.2	1.1	0.5	2.3	0.4	2.0	3.4	13.7	5.1	17.1	4.7	15.0	1.5	10.7	2.8	21.5	2.4	17.9	0.2	1.5	0.1	1.1	0.1	1.1	10.3	33.3	3.4	8.9	2.8	7.5
Mato Grosso do Sul	0.3	1.4	0.4	3.1	0.4	2.8	3.3	9.9	4.4	12.8	4.0	11.3	1.4	8.1	2.2	15.4	1.8	13.1	0.1	1.2	0.1	0.8	0.1	0.7	11.2	37.0	2.9	7.8	2.3	6.6
Minas Gerais	0.1	0.7	0.2	1.4	0.2	1.3	2.2	7.5	3.9	14.8	3.6	13.2	1.0	7.3	1.3	10.4	1.2	9.1	0.2	0.9	0.1	0.6	0.1	0.6	10.9	28.9	2.8	7.3	2.4	6.3
Pará	0.1	0.5	0.3	1.2	0.3	1.1	1.3	5.1	1.7	7.3	1.7	6.7	0.8	4.7	1.8	13.1	1.6	11.7	0.1	0.7	0.1	0.7	0.1	0.7	10.6	36.9	3.8	13.6	3.2	11.9
Paraíba	0.2	1.0	0.2	1.1	0.2	1.1	3.1	13.2	2.5	12.5	2.4	11.5	1.9	7.6	2.0	20.4	1.8	18.4	0.2	0.8	0.1	0.7	0.1	0.6	8.1	23.4	3.1	10.0	2.5	8.8
Paraná	0.2	0.9	0.3	2.3	0.3	2.1	2.9	9.4	4.8	16.2	4.4	14.9	1.3	7.9	1.9	13.8	1.6	12.1	0.2	1.0	0.1	0.7	0.1	0.7	12.7	40.9	3.4	10.1	2.7	8.8
Pernambuco	0.1	0.6	0.2	1.5	0.2	1.4	1.9	6.7	2.6	10.2	2.4	9.7	0.8	3.5	1.8	18.4	1.6	16.8	0.2	0.8	0.1	0.6	0.1	0.6	11.2	38.6	3.4	10.9	2.8	10.1
Piauí	0.3	2.2	0.3	2.7	0.3	2.5	2.9	11.2	2.7	9.8	2.6	8.6	2.5	11.9	3.3	35.1	3.0	29.3	0.1	1.1	0.1	0.8	0.1	0.8	7.8	23.8	2.9	8.8	2.4	7.5
Rio de Janeiro	0.1	0.9	0.2	1.4	0.2	1.3	1.7	5.8	2.2	7.2	2.1	6.9	1.0	5.3	1.2	9.0	1.1	8.2	0.1	0.6	0.1	0.5	0.1	0.5	13.0	40.7	3.6	10.0	3.2	9.2
Rio Grande do Norte	0.1	0.7	0.2	1.4	0.2	1.3	2.5	11.8	2.3	9.2	2.1	9.1	1.1	5.8	1.6	14.6	1.4	13.7	0.2	0.8	0.1	0.6	0.1	0.6	8.0	27.6	2.2	7.2	1.8	6.8
Rio Grande do Sul	0.1	0.5	0.2	1.2	0.2	1.1	2.7	10.3	3.1	11.0	3.0	10.3	0.9	5.9	1.2	8.4	1.2	7.7	0.1	0.7	0.1	0.5	0.1	0.5	7.1	20.8	2.5	6.5	2.2	5.9
Rondônia	0.2	1.0	0.4	2.4	0.4	2.2	3.0	12.4	3.8	15.3	3.6	14.2	1.7	10.5	3.0	22.6	2.6	20.8	0.2	1.3	0.1	1.2	0.1	1.2	11.9	38.5	3.5	10.7	2.8	9.4
Roraima	0.5	3.1	0.8	5.5	0.7	5.4	4.3	14.1	4.6	12.8	4.5	12.6	2.3	12.9	2.6	19.8	2.6	18.6	0.1	0.9	0.1	0.6	0.1	0.6	9.5	36.0	2.6	8.2	2.3	7.6
Santa Catarina	0.3	1.5	0.5	2.6	0.4	2.4	5.3	20.0	4.9	15.2	4.7	14.1	1.3	10.0	1.9	14.3	1.8	13.0	0.1	0.8	0.1	0.4	0.1	0.4	12.6	36.7	2.7	6.9	2.3	6.3
São Paulo	0.1	0.8	0.2	1.2	0.2	1.1	1.7	6.8	2.1	7.8	2.0	7.0	1.0	7.2	1.2	8.7	1.1	7.7	0.2	0.8	0.1	0.4	0.1	0.4	12.5	39.1	2.7	7.8	2.3	6.7
Sergipe	0.1	1.3	0.1	2.3	0.1	2.1	3.3	12.0	3.0	11.4	2.8	10.7	1.4	6.8	1.8	22.3	1.7	19.7	0.2	1.3	0.1	0.9	0.1	0.9	11.5	42.3	2.8	9.6	2.4	8.7
Tocantins	0.5	2.4	0.5	3.0	0.5	3.0	6.9	23.4	5.5	18.6	5.1	17.7	3.0	10.6	3.3	21.0	3.0	20.3	0.2	1.8	0.1	1.4	0.1	1.3	7.7	24.0	2.9	9.4	2.5	8.5
