# Supplementary material for: Road traffic injuries and deaths and the achievement of UN Sustainable Development Goals in Brazil: results from the Global Burden of Disease Study, 1990 to 2019
Source: Rev Soc Bras Med Trop. 2022 Jan 28;55(Suppl 1):e0261-2021. doi: 10.1590/0037-8682-0261-2021 (PMC9038143; doi:10.1590/0037-8682-0261-2021)
Supplement: Supplementary file 4 [file 1678-9849-rsbmt-55-s01-e0261-2021-supp4.pdf]

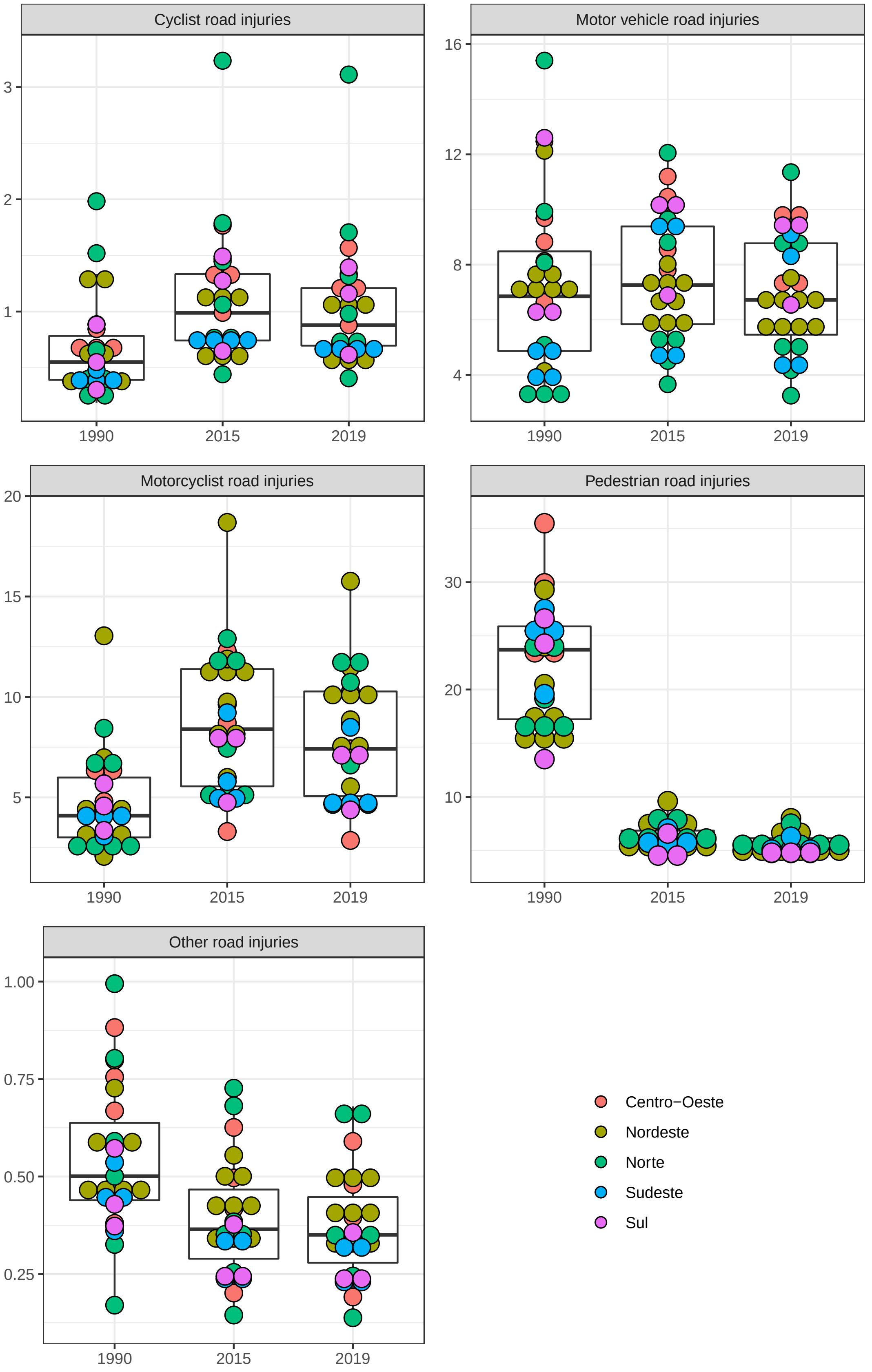

Supplementary Material 3 - Age-standardized r[illegible]rtality rates by type of road transport per 100,000 inhabitants, both sexes, according to the region of E[illegible]zil and year, GBD 2019.
